# Elucidating the Consequences of Heparan Sulfate Binding by Heparanase 2

**DOI:** 10.3389/fonc.2020.627463

**Published:** 2021-01-29

**Authors:** Miriam Gross-Cohen, Sari Feld, Gil Arvatz, Neta Ilan, Israel Vlodavsky

**Affiliations:** Technion Integrated Cancer Center, Rappaport Faculty of Medicine, Technion, Haifa, Israel

**Keywords:** heparanase, heparanase 2, heparan sulfate, adhesion, migration, scattering

## Abstract

Unlike the intense research effort devoted to exploring the significance of heparanase in human diseases, very little attention was given to its close homolog, heparanase 2 (Hpa2). The emerging role of Hpa2 in a rare autosomal recessive congenital disease called urofacial syndrome (UFS), clearly indicates that Hpa2 is not a pseudogene but rather a gene coding for an important protein. Hpa2 lacks the heparan sulfate (HS)-degrading activity typical of heparanase, yet exhibits high affinity to HS, affinity that is 10-fold higher than that of heparanase. The consequences of this high-affinity interaction of Hpa2 with plasma membrane HSPG has not been explored yet. Here, we used highly purified Hpa2 protein to examine this aspect. We provide evidence that cells adhere to and spread on dishes coated with Hpa2. We also show that cell migration is attenuated markedly by exogenous addition of Hpa2 to primary and transformed cells, a function that agrees with the anti-cancer properties of Hpa2. Interestingly, we found that exogenous addition of Hpa2 also disrupts the morphology of cell colonies, resulting in cell scattering. This implies that under certain conditions and experimental settings, Hpa2 may exhibit pro-tumorigenic properties. We further developed a panel of anti-Hpa2 monoclonal antibodies (mAb) and show that these properties of Hpa2 are prevented by some of the newly-developed mAb, thus providing new molecular tools to better appreciate the significance of Hpa2 in health and disease.

## Introduction

Heparanase is a unique enzyme due to its endoglycosidase activity, capable of cleaving heparan sulfate (HS) side chains of heparan sulfate proteoglycans (HSPG). HSPG are highly abundant in the extracellular matrix (ECM) and assist to assemble the major protein constituents of the ECM (i.e., laminin, fibronectin, collagen-IV, etc.) into a three-dimensional, non-soluble, thick matrix that provides structural support and biochemical cues to many cell types. Cleavage of HS by heparanase thus results in remodeling of the ECM. These structural and biochemical alterations are expected to exert a profound impact on cell behavior including, among others, cell differentiation, proliferation, migration, and invasion. The latter is most often associated with increased metastatic capacity of tumor cells and augmented entry of inflammatory cells (i.e., T-cells, macrophages, NK-cells) to sites of inflammation (1–3). Heparanase also cleaves HSPG on the cell surface (i.e., syndecans), affecting their ability to function as co-receptors in signaling pathways. In addition, cleavage of the HS side chains of syndecan-1 augments the shedding of this proteoglycan from the surface of myeloma cells, leading to a more aggressive disease (4, 5). This, and many other mechanisms utilized by heparanase to promote tumorigenesis (3, 5–9), have turned this enzyme into a promising drug target and heparanase inhibitors are currently being evaluated in clinical trials as anti-cancer (10, 11) and anti-viral (12) drugs.

Heparanase 2 (Hpa2) is a close homolog of heparanase; it shows an overall identity of 40% and sequence resemblance of 59% with heparanase, including conservation of residues critical for heparanase enzymatic activity (Glu_225_ and Glu_343_) (13). Hpa2 nonetheless lacks the HS-degrading activity typical of heparanase (14). Like heparanase, Hpa2 is secreted and interacts with cell membrane syndecans. Unlike heparanase, Hpa2 is not internalized into endocytic vesicles but rather is retained on the cell membrane for a relatively long period of time (14). The reason for the failure of Hpa2 to get internalized is not known, but may be due to its high affinity to HS, affinity that is 10-fold higher than that of heparanase (14). The consequences of this high-affinity interaction of Hpa2 with plasma membrane HSPG have not been explored yet. Here, we used highly purified Hpa2 protein to examine this aspect. We provide evidence that cells adhere to and spread on dishes coated with Hpa2. We also show that cell migration is attenuated markedly by exogenous addition of Hpa2 to primary and transformed cells, a function that agrees with the anti-cancer properties of Hpa2 (14–17). Interestingly, we found that exogenous addition of Hpa2 also disrupts the morphology of cell colonies, resulting in cell scattering. This implies that under certain conditions and experimental settings, Hpa2 may exhibit pro-tumorigenic properties. Importantly, attenuation of cell migration and cell scattering by Hpa2 appears to be HS-dependent and was attenuated by heparin. We further developed a panel of anti-Hpa2 monoclonal antibodies (mAb) and show that these properties of Hpa2 are prevented by some of the newly-developed mAb, thus providing new molecular tools to better appreciate the significance of Hpa2 in health and disease.

## Materials and Methods

### Antibodies and Reagents

All reagents were purchased from Sigma unless specified otherwise. Anti-Hpa2 monoclonal antibodies were raised against Hpa2 protein purified from the conditioned medium of Hpa2-transfected HEK293 cells, essentially as described (14, 18). Briefly, Balb/C mice were immunized with the full-length Hpa2 protein. Hybridomas were obtained by routine procedures and were selected by ELISA using Hpa2 for coating. Several hybridomas that reacted positively with Hpa2 were selected for further characterization. Performance of the newly developed monoclonal antibodies is summarized in **Table 1**. Anti-syndecan-1 (sc-5632), anti-syndecan-4 (sc-12766), anti-Erk (sc-94), anti-phospho-Erk (sc-7383), anti-focal adhesion kinase (FAK; sc-932), and anti-Akt (sc-5298) antibodies were purchased from Santa Cruz Biotechnology (Santa Cruz, CA). Anti-phospho-Akt and anti-phospho-FAK antibodies were purchased from Cell Signaling (Danvers, MA). Anti-vinculin and anti-actin antibodies were purchased from Sigma.

**Table 1:** Performance of anti-Hpa2 monoclonal antibodies.

Hybridoma	Immuno-blotting	IF	IP	Neutralizing:		
				Cell adhesion	Cell migration	Cell scattering
**1c7**	+	NT	–	no	no	yes
**20c5**	++	++	++	yes	yes	yes
**21b9**	+	NT	+	NT	yes	no
**33a19**	–	NT	+	yes	no	no
**2B9**	++	NT	++	NT	NT	yes
**5G4**	+	–	NT	NT	NT	NT
**6D11**	++	++	NT	yes	no	NT
**6D12**	+	+	NT	yes	no	NT
**6F7**	++	–	NT	NT	NT	NT
**7A6**	+	–	NT	NT	NT	NT
**10G3**	+	+	NT	NT	NT	no

IF, Immunofluorescent staining.

IP, Immunoprecipitation.

NT, Not tested.

-: does not work.

+: work.

++: work well.

### Cells and Cell Culture

U-87 MG glioma, SIHN-013 laryngeal carcinoma, A-549 lung adenocarcinoma and HEK-293 cells have been described previously (14, 15, 19, 20) and were grown in Dulbecco’s modified Eagle’s medium (Biological Industries, Beit Haemek, Israel) supplemented with 10% FCS and antibiotics. Human (5637, RT4) and mouse (MBT2-t50) bladder carcinoma, and U266 B-lymphoma cells have been described previously (16, 21–24) and were grown in RPMI-1640 supplemented with 10% FCS. Human umbilical vein-derived endothelial cells (HUVEC) were cultured in M199 medium containing 20% FCS supplemented with 50 µg/ml bovine hypothalamus Endothelial Mitogen (ECGS) BT-203 (Biomedical Technologies, MA, USA) and 20 µg/ml heparin. Cells were plated on dishes pre-incubated with 0.2% gelatin for 1 h at 37°C, as described (25). HUVEC were not used beyond passage eight. Porcine aortic endothelial (PAE) cells were cultured in F-12 medium containing 10% FCS, glutamine, and antibiotics, as described previously (26). MCF10A cells were kindly provided by Dr. Yosef Yarden (Weizmann Institute of Science, Rehovot, Israel) and were maintained in DMEM-F12 medium (1:1) supplemented with 0.1 μg/ml cholera toxin, 0.02 μg/ml epidermal growth factor, 10 μg/ml insulin, 0.5 μg/ml hydrocortisone, 100 U/ml penicillin, 100 μg/ml streptomycin, and 5% horse serum, as described (27).

### Cell Adhesion

Twenty-four–well plates were coated with 10 µg/ml of BSA, fibronectin, heparanase and Hpa2 for 18 h at 4°C (or 1 h at 37°C). Cells were plated in medium supplemented with 10% FCS and their adhesion, morphology and spreading properties were observed. After 1 h, cells were washed, fixed with 4% PFA, and photographed with or without crystal violet (0.5%) staining. To examine the signaling associated with cell adhesion to heparanase/Hpa2, cells were plated in the absence or presence of heparin (50 µg/ml) or the indicated anti-Hpa2 monoclonal antibody (30 µg/ml). For biochemical analyses, cell extracts were prepared and subjected to immunoblotting with the indicated antibody or were fixed with cold methanol and subjected to immunofluorescent staining, as described (14, 28).

### Cell Migration

Cells were plated in an ibidi wound scratch apparatus (Planegg, Germany) according to the manufacturer’s instructions (Culture-Insert; ibidi), as described (16). Briefly, cells (3x10^4^ in 0.1 ml) were seeded inside the apparatus. Once confluent, the inserts were removed, leaving a well-defined gap. Cell cultures were washed and changed to serum-free medium or medium containing 2% serum, and migration into the defined cell-free gap was inspected in the absence or presence of 10 µg/ml purified Hpa2. Cell migration into the gap was examined under light microscope and images were taken at the indicated time after Hpa2 addition without or with heparin (50 µg/ml) or the indicated mAb (30 µg/ml).

### Cell Scattering

Cells were plated in 12-well plates and were grown until discrete colonies were established. The indicated concentration of purified heparanase or Hpa2 proteins were then added in serum-free medium, alone or together with heparin (50 µg/ml), or the indicated anti-Hpa2 monoclonal antibody, and the morphology of cell colonies was observed after 24 h.

### Protein Extraction and Immunoblotting

Preparation of cell lysates and immunoblotting was performed essentially as described (14–16). Briefly, cell cultures were pretreated with 1 mM orthovanadate for 10 min at 37°C, washed twice with ice-cold PBS containing 1 mM orthovanadate and scraped into lysis buffer (50 mM Tris-HCl, pH 7.4, 150 mM NaCl, 0.5% NP-40, 1 mM orthovanadate, 1 mM PMSF) containing a cocktail of proteinase inhibitors (Roche). Total cellular protein concentration was determined by the BCA assay according to the manufacturer’s instructions (Pierce, Rockford, IL). Fifty μg of cellular protein were resolved on SDS polyacrylamide gel, and immunoblotting was performed, as described (14–16).

### Flow Cytometry

Cells were detached with 0.5 mM EDTA, centrifuged at 1000 rpm for 4 min, washed 3 times with washing buffer (PBS containing 0.1% FCS) and counted. Cells (0.5x10^6^) were re-suspended in PBS containing 1% FCS and incubated with anti-syndecan-1 or -4 antibodies for 30 min on ice in light protected tubes. Negative control was incubated without the primary antibody. Cells were then washed 3 times with the above washing buffer and incubated as above with 488-conjugated secondary antibody, washed, and analyzed using a CyAn fluorescent activated cell sorter (Beckman coulter) and Summit software, as described (28, 29).

## Results

### Hpa2 Promotes HS-Dependent and -Independent Cell Adhesion

**Figure 1:**
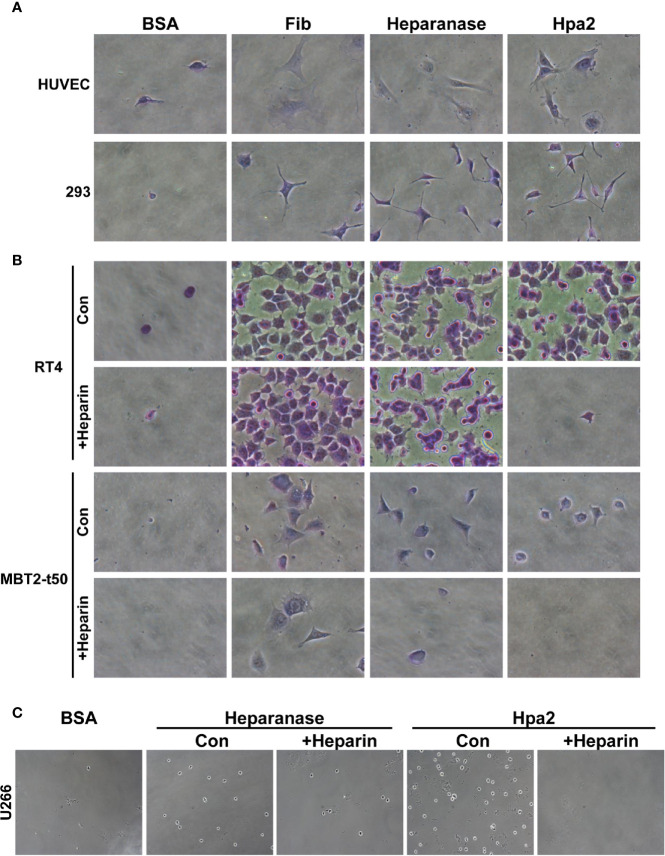
Hpa2 promotes cell adhesion. Twenty-four–well plates were coated with 10 µg/ml of the indicated protein (18 h, 4°C), followed by blocking with BSA for 1 h at room temperature. The indicated cell type was then plated in growth medium and cell adhesion and spreading properties were observed. After 1 h, cells were washed, fixed, and photographed. Where indicated, heparin (50 µg/ml) was added together with the cells upon plating. **(A)** HUVEC (upper panels) and HEK 293 (lower panels). **(B)** RT4 human bladder (upper and second panels) and MBT2-t50 murine bladder carcinoma cells (third and fourth panels). **(C)** U266 myeloma cells. Shown are representative images at x50 **(A, B)**, x25 **(C)** (original magnification).

**Figure 2:**
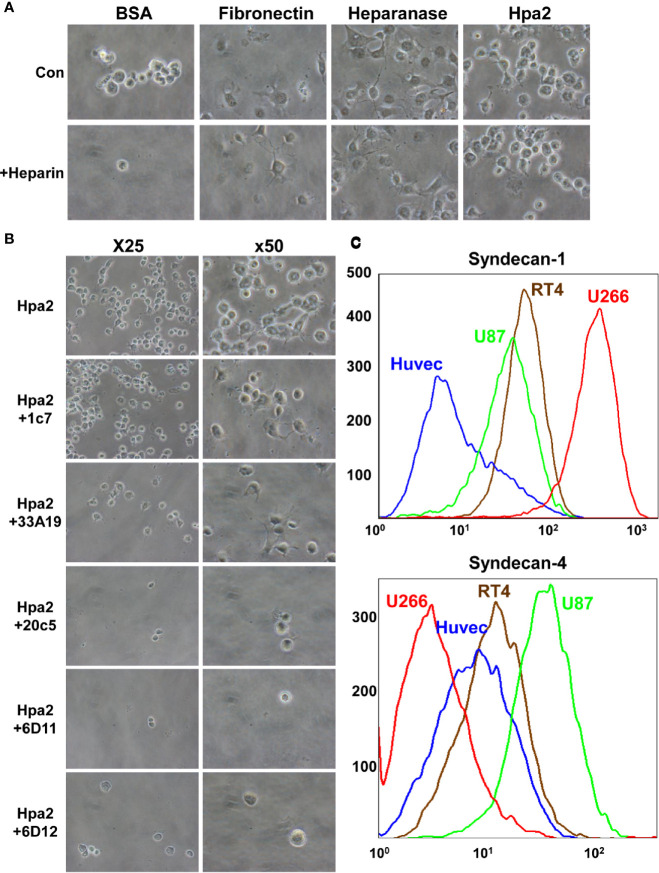
**(A)** Adhesion of U87 glioma cells to Hpa2 is heparin-independent. U87 cells were plated on dishes coated with the indicated protein in the absence (upper panels) or presence (lower panels) of heparin (50 µg/ml). After 1 h cells were washed, fixed, and photographed. Shown are representative images at x50 (original magnification). **(B)** Inhibition of cell adhesion and spreading by anti-Hpa2 mAbs. U87 glioma cells were plated on Hpa2-coated dishes without (upper panel, Hpa2) or with the indicated mAb (30 µg/ml). Cells were also incubated with heparin (50 µg/ml). Cells were allowed to adhere and spread for 1 h, washed and fixed. Shown are representative images at x25 and x50 (original magnification). **(C)** FACS analysis. HUVEC, U87, RT4, and U266 cells were subjected to FACS analyses applying anti-syndecan-1 (upper panel) and anti-syndecan-4 (lower panel) antibodies.

We have reported previously that Hpa2 lacks the capacity to cleave HS, the hallmark of heparanase, but retains the ability to bind HS with high affinity (14). The consequences of this interaction have not been elucidated yet. To examine this aspect we purified Hpa2 to high purity levels (**Supplementary Figure 1A** ****) and evaluated the capacity of cells to adhere to dishes coated with Hpa2. This was envisioned based on previous reports that implicate latent heparanase and its HS-binding domains in cell adhesion (30–33). BSA and fibronectin were used as negative and positive controls, respectively, along with purified heparanase (34). We found that primary human umbilical vein endothelial cells (HUVEC; **Figure 1A**, second panels) and non-transformed HEK-293 cells (**Figure 1A**, upper panels) adhere and spread on Hpa2-coated dishes to a magnitude comparable to fibronectin and heparanase. Similarly, bladder carcinoma cells RT4 and MBT2-t50 adhered efficiently to dishes coated with Hpa2, heparanase, and fibronectin (**Figure 1B**, upper and third panels). Notably, adhesion of the bladder carcinoma cells to Hpa2-, and to a lesser extent to heparanase-coated dishes was attenuated markedly by heparin (**Figure 1B**, second and fourth panels). Cell adhesion to fibronectin was not affected by heparin (**Figure 1B**). Furthermore, heparanase and Hpa2 supported the adhesion of RPMI8266 (**Supplementary Figure 1B**) and U266 myeloma cells that normally grow in suspension, and this was similarly abrogated by heparin (**Figure 1C**), suggesting that the pro-adhesive properties of Hpa2 are mediated primarily by HS. Likewise, Hpa2 promoted the adhesion and spreading of U87 glioma cells (**Figure 2A**, upper panels). In striking contrast, nonetheless, adhesion of U87 cells to dishes coated with heparanase or Hpa2 was not affected by heparin (**Figure 2A**, lower panels). In order to ascertain this result we examined the capacity of a panel of newly developed anti-Hpa2 mAbs (**Table 1**) to interfere with the adhesion of U87 cells to dishes coated with Hpa2. We found that U87 cell adhesion was attenuated prominently by anti-Hpa2 mAb 20c5, 6d11, and 6d12 (**Figure 2B**, lower panels) while other monoclonal antibodies (i.e., 1c7, 33a19) were less effective (**Figure 2B**; **Table 1**). These results critically support the notion that Hpa2 exerts HS-dependent and -independent pro-adhesive properties, depending on the cell type.

Given that adhesion of RT4 cells to Hpa2 was practically prevented by heparin whereas adhesion of U87 cells was heparin-independent (**Figures 1B** and **2A**), we examined the occurrence of HSPG on their cell membrane. FACS analyses revealed that U266 cells express the highest levels of syndecan-1 (**Figure 2C**, upper panel), as would be expected for myeloma cells, whereas U87 cells express high levels of syndecan-4 (**Figure 2C**, lower panel). Seemingly, RT4 and U87 cells exhibited comparable levels of syndecan-1 and syndecan-4, suggesting that their adhesion to Hpa2 and its HS-dependency involve a more complex mechanism beyond mere interaction with HS.

**Figure 3:**
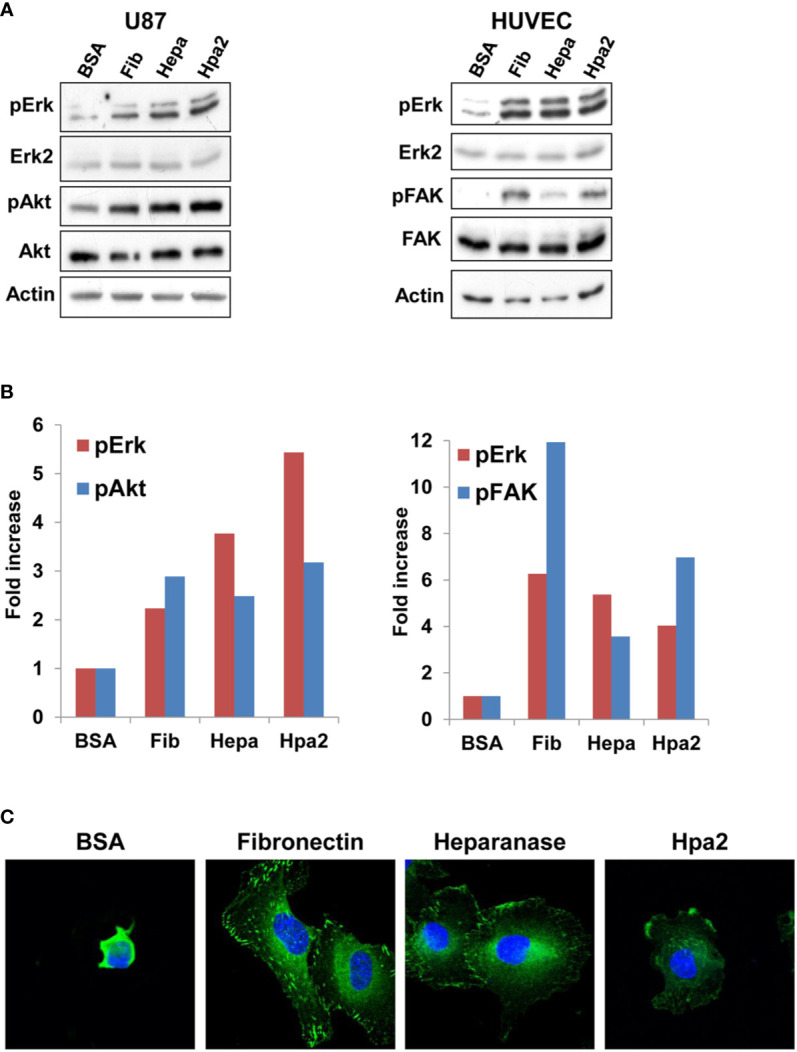
Signaling. U87 (**A,** left) and HUVEC (**A,** right) cells were plated on dishes coated with the indicated protein for 1 h. Cell extracts were then prepared from adherent and floating cells and subjected to immunoblotting applying antibodies directed against pErk (upper panel), Erk2 (second panels), pAkt (left third panel), Akt (left fourth panel), pFAK (right third panel), FAK (right fourth panel), or Actin (lower panels). Densitometry analysis of Erk, Akt, and FAK phosphorylation is shown graphically in **(B)**. **(C)** Immufluorescent staining. HUVEC were plated on dishes coated with the indicated protein for 1 h. Cells were then fixed with 4% PFA, followed by treatment with 0.5% triton x100 for 1 min. Cells were then subjected to immunofluorescent staining applying anti-vinculin antibody (green). Nuclear counterstaining is shown in blue. Note, focal adhesions in cells that adhered to fibronectin, and less so in cells that adhered to heparanase or Hpa2.

To examine the signaling pathways associating with cell adhesion to Hpa2 we next plated U87 and HUVEC cells on dishes coated with fibronectin, heparanase, or Hpa2 and cell extracts were subjected to immunoblotting. We found that cell adhesion to fibronectin was accompanied by increased phosphorylation of Erk (Fib; **Figure 3A**, upper panels), as would be expected. Interestingly, a comparable increase in Erk phosphorylation was noted in cells plated on heparanase and Hpa2 (**Figure 3A**, upper panels). Similarly, cell adhesion to Hpa2 was associated with increased phosphorylation of Akt (**Figure 3A**, left third panel) and focal adhesion kinase (FAK; **Figure 3A**, right third panel), signaling molecules highly implicated in integrin-mediated cell adhesion (35). Immunofluorescent staining of vinculin showed typical focal contacts in cells plated on fibronectin (Fib; **Figure 3C**). In contrast, cells plated on heparanase or Hpa2 appeared less spread and exhibited very few and immature focal contacts (**Figure 3C**). This suggests that induction of Erk, Akt and FAK phosphorylation by Hpa2 involves mechanism other than the classical integrin-mediated focal adhesion (35, 36), likely signaling by syndecans (37–39).

### Hpa2 Attenuates Cell Migration

**Figure 4:**
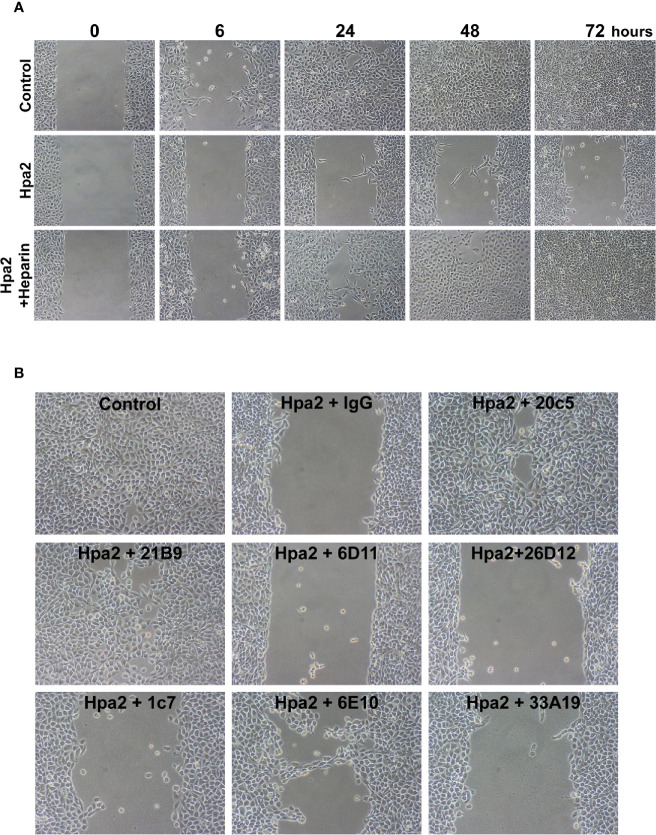
Hpa2 attenuates cell migration. 5637 bladder carcinoma cells were plated in ibidi cell migration inserts apparatus (Planegg, Germany) until confluent. The barriers were then removed, cell cultures were washed and changed to serum-free medium, and migration into the defined cell-free gap was inspected in the absence (Control) or presence of purified Hpa2, Hpa2 plus heparin (50 µg/ml; **A**), and in the presence of Hpa2 and the indicated anti-Hpa2 mAb (30 µg/ml; **B**). Shown are representative photomicrographs taken before (0), and 6, 24, 48, and 72 h after the addition of Hpa2 in the absence (Hpa2) and presence of heparin, control mouse IgG or the anti-Hpa2 mAbs. Note that cell migration and wound closure is attenuated prominently by exogenous Hpa2, and this effect is reversed by some of the anti-Hpa2 mAbs, and heparin. Shown are representative images at x50 (original magnification).

Applying the ibidi wound scratch apparatus we found that exogenous addition of Hpa2 attenuates the migration of 5637 (**Figure 4A**, upper vs middle panels), primary MCF10A human breast (**Supplementary Figure 1C**), primary porcine endothelial (PAEC; **Supplementary Figure 1D**, upper panels), head and neck carcinoma SINH-013 (**Supplementary Figure 1D**, middle panel), and lung carcinoma A549 (**Supplementary Figure 1D**, lower panel) cells. Importantly, attenuation of cell migration by Hpa2 was compromised by heparin (**Figure 4A**, lower panels), implying that like cell adhesion, this function of Hpa2 is also HS-dependent. We further examined our panel of anti-Hpa2 mAbs in the cell migration assay. We found that attenuation of 5637 cell migration by Hpa2 was abolished by mAbs 20c5 and 21B9 while other mAbs (i.e., 6D11, 6D12, 1c7, 6E10, 33A10) were not effective (**Figure 4B**; **Table 1**), laying more confidence that this effect is mediated by Hpa2.

### Hpa2 Promotes Cell Colonies Dissociation

**Figure 5:**
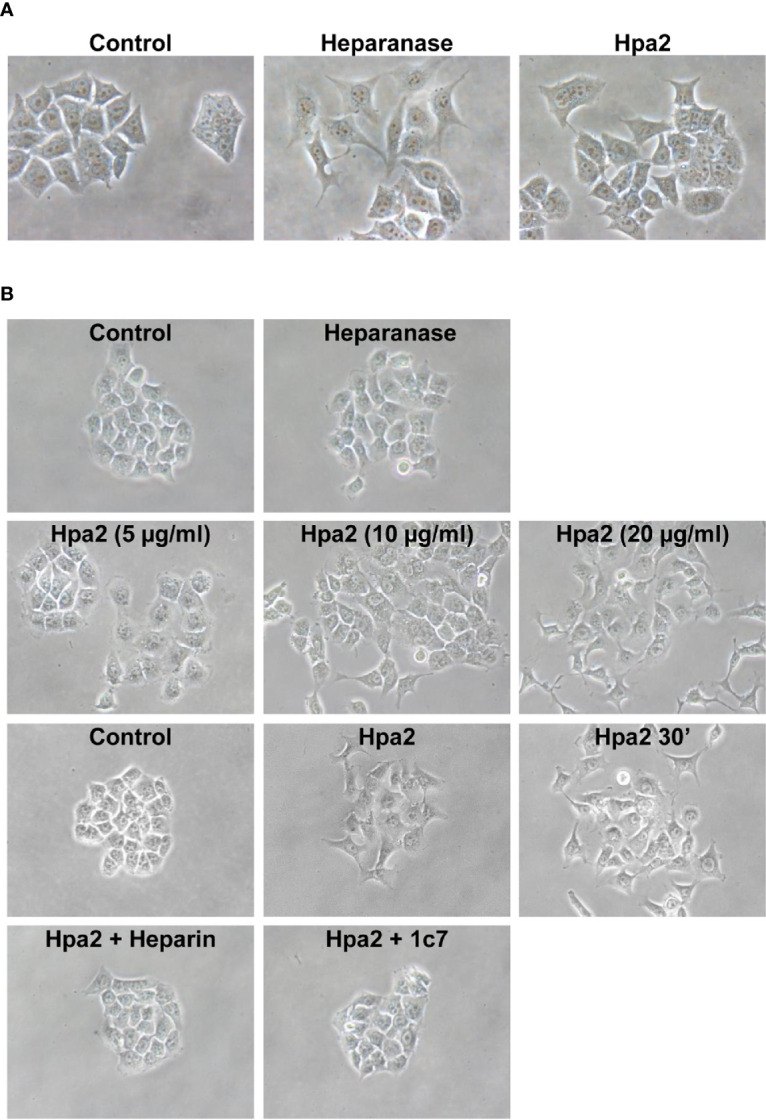
Cell scattering. **(A)** RT4 bladder carcinoma cells were allowed to grow for 2–3 days until cell colonies were formed. Heparanase or Hpa2 (10 µg/ml) were then added and colonies morphology was examined after 24 h vs. control (Control) untreated cells. **(B)** SINH-013 laryngeal carcinoma cells were similarly treated with heparanase or the indicated concentration of Hpa2 (second panels), or were treated with Hpa2 for 30 min; Medium was then removed, the cell culture washed and replaced with fresh medium without Hpa2 (third panels, Hpa2 30’). Colony morphology and cell scattering was examined after 24 h vs. cultures untreated (Control) or treated with Hpa2 (third panels). Cell scattering was similarly examined in cell cultures treated with Hpa2 in the presence of heparin (50 µg/ml; Hpa2+Heparin) or mAb 1c7 (30 µg/ml; Hpa2+1c7) (lower panels). Shown are representative images at x100 (original magnification).

**Figure 6:**
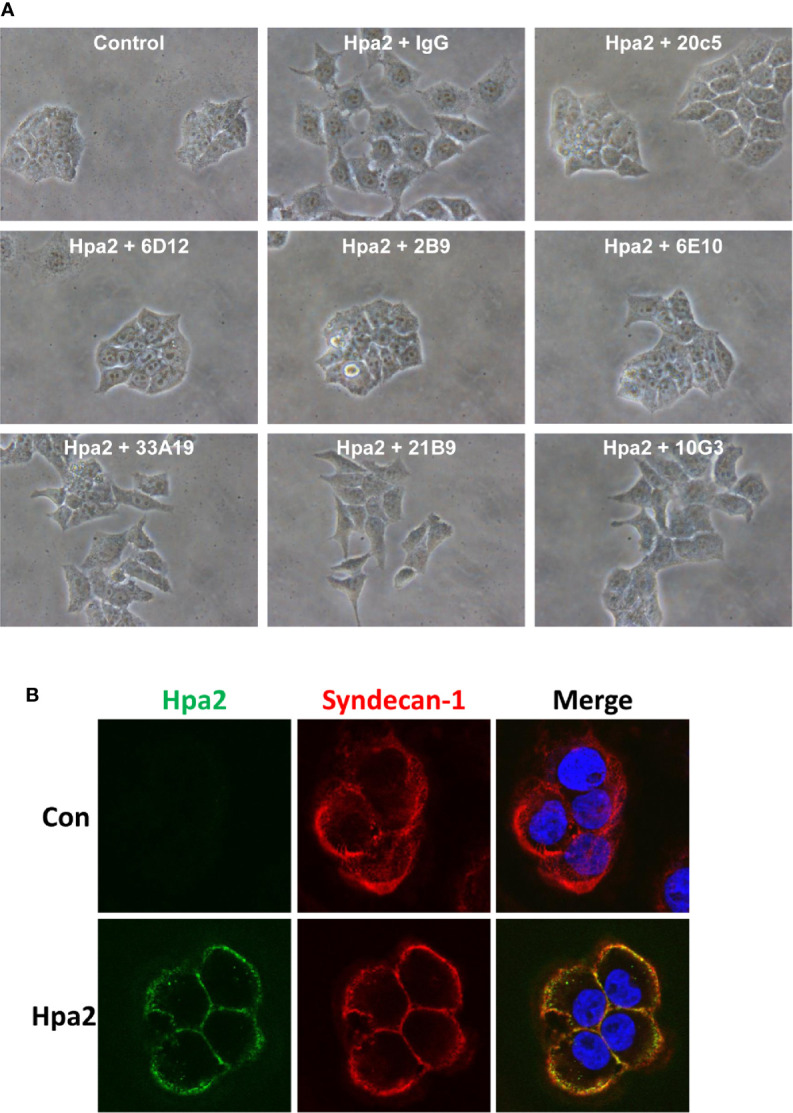
**(A)** Anti-Hpa2 mAbs neutralize the scattering capacity of Hpa2. RT4 cells were allowed to establish colonies and were then treated with Hpa2 (10 µg/ml) in the presence of control mouse IgG or the indicated anti-Hpa2 mAb (30 µg/ml). Colony morphology was examined after 24 h. Shown are representative images at x100 (original magnification). **(B)** Immunofluorescent staining. SIHN-013 cells were left untreated (Control; upper panels) or were incubated with Hpa2 (5 µg/ml) for 30 min (lower panels). Cells were then washed, fixed, and subjected to immunofluorescent staining applying anti-Hpa2 (20c5; green) and anti-syndecan-1 (red) antibodies. Merge images are shown in the right panels. Note co-localization of Hpa2 and syndecan-1 on the cell membrane.

We have next examined the behavior of RT4 bladder carcinoma cells following the addition of our purified heparanase and Hpa2 proteins to the cell culture medium. RT4 cells grow in typical well-organized colonies (**Figure 5A**, Control). Notably, the organization of cell colonies was disrupted following the addition of heparanase. Unexpectedly, organization of cell colonies was disrupted also by Hpa2, leading to a scattered morphology (**Figure 5A**). Likewise, dissociation of cell colonies and induction of cell scattering were evident in non-transformed MDCK cells following exogenous addition of heparanase and Hpa2 (**Supplementary Figure 1E**). In laryngeal SINH-013 carcinoma cells, scattering and dissociation of cell colonies by Hpa2 were dose-dependent (**Figure 5B**, second panels) and exposure to Hpa2 for only 30 min was sufficient to elicit colony dissociation (**Figure 5B**, 30’). Furthermore, cell scattering by Hpa2 was prevented by heparin (**Figure 5B**, Hpa2+Heparin) and by mAb 1c7 (**Figure 5B**, Hpa2+1c7), a mAb shown previously to target the HS-binding domain of Hpa2 (15). Similarly, dissociation of RT4 colonies by Hpa2 was abrogated by anti-Hpa2 mAb 20c5, 6D12, 2B9, and 6E10 (**Figure 6A** upper and middle panels) while other mAbs had no effect (i.e., 33A19, 21B9, 10G3; **Figure 6A**, lower panels) (**Table 1**).

As noted previously (14), Hpa2 appeared tethered to the cell membrane following exogenous addition, co-localizing with syndecan-1 (**Figure 6B**), and failed to get internalized, suggesting that the effect of Hpa2 is initiated primarily at the cell membrane.

## Discussion

Unlike the intense research effort devoted to exploring the significance of heparanase in human diseases (40), very little attention was given to its close homolog, heparanase 2 (Hpa2). The emerging role of Hpa2 in a rare autosomal recessive congenital disease called urofacial syndrome (UFS) (41–43), clearly indicates that Hpa2 is not a pseudogene but rather a gene coding for an important protein. Most recently, it was reported that Hpa2 levels are prominently decreased in critically ill Covid-19 patients, resulting in high heparanase to Hpa2 ratio (44). Accordingly, shedding of syndecan-1 was increased, while the thickness of the endothelial glycocalyx that contributes to the maintenance of vascular homeostasis was markedly reduced in Covid-19 patients (44). This may suggest that Hpa2 functions to preserve and protect the vasculature, in line with the notion that Covid-19 is a vascular disease (45). This may turn very important in other diseases that involve disruption of the glycocalyx such as sepsis (46). Indeed, Hpa2 was found to protect the vascular endothelium from LPS and in conditions of sepsis. Hpa2-overexpressing endothelial cells were protected against LPS-mediated loss of cell-cell contacts and administration of purified Hpa2 to mice resulted in decreased plasma levels of TNFα and IL-6 after LPS injection (47).

In line with this notion is the observed decrease in Hpa2 expression in cancer patients (14, 16, 17, 48, 49), implying that Hpa2 not only protects the vascular endothelium but also the epithelium. Indeed, cancer patients that express high levels of Hpa2 were diagnosed mostly as low-grade, expressed higher levels of cytokeratins, and survived for a longer time (14–17, 50, 51). Furthermore, overexpression of Hpa2 resulted in smaller tumors endowed with reduced vascular density (blood and lymphatic) (15), thus expending the role of Hpa2 in vascular biology.

The capacity of Hpa2 to inhibit heparanase enzymatic activity (14) implies that Hpa2 functions as an endogenous inhibitor of heparanase, leading to evaluation of heparanase to Hpa2 ratio as an important parameter in diseases (44). Hpa2, nonetheless, can function in a manner that seems heparanase-independent. A prime suspect underlying this aspect is the capacity of Hpa2 to interact with HS with high affinity (14), resulting in clustering of cell membrane HSPG (i.e., syndecans) and likely activation of signaling cascades. Thus, while Hpa2 lacks HS-degrading activity typical of heparanase, its ability to bind HS is preserved. In fact, Hpa2 exhibits a 10-fold higher affinity to HS than heparanase (14), but the biological significance of this interaction was unclear. Utilizing a highly purified Hpa2 protein we show here, for the first time, that Hpa2 directs cell adhesion and motility and that these effects, to a large extent, are HS-dependent. Our results demonstrate that Hpa2 attenuates the migration of non-transformed (PAEC, MCF10A) and malignant (5637, SINH-013, A549) cells (**Figure 4**; **Supplementary Figures 1C, D**). Importantly, attenuated cell migration by Hpa2 was reversed once heparin was added together with Hpa2 (**Figure 4A**), preventing its interaction with cell membrane HSPG (14). Possibly, the added Hpa2 decreases the heparanase to Hpa2 ratio and antagonizes the pro-migratory effect of heparanase (25, 33), while heparin tilts this ratio back in favor of heparanase. However, given that heparin is a strong inhibitor of heparanase enzymatic activity (52), the increased cell migration following addition of heparin is not likely to be attributed to heparanase. Instead, exogenous Hpa2 likely elicits signaling from the cell membrane that affects cell motility which is prevented by heparin. The nature of the signal transduction involved is unclear but may be related to the signaling evoked by Hpa2-mediated cell adhesion (**Figure 3**).

Previous studies have shown that heparanase not only promotes cell invasion, associated with its well-established pro-metastatic properties but also promotes cell adhesion and migration. Unlike the pro-invasive properties of heparanase, increased cell adhesion and migration appeared enzymatic activity-independent (25, 30, 31, 53). Instead, increased cell adhesion and spreading by heparanase likely involves signaling aspects due, in part, to clustering of cell membrane HSPG. This was concluded because cell adhesion and spreading was promoted also by a peptide derived from the HS-binding domain (KKDC) of heparanase, involving activation of Rac (31). Similarly, primary and transformed cells readily adhere to dishes coated with Hpa2 in an HS-dependent manner that was inhibited by heparin (**Figures 1B, C**). Curiously, adhesion of some cell lines (i.e., U87 and HUVEC) to Hpa2 was not attenuated by heparin (**Figures 2A, B** **and** data not shown), which cannot be explained simply by the abundance of syndecan-1 and -4 on the cell membrane (**Figure 2C**). This suggests a more complex mechanism that possibly involves other Hpa2-binding protein(s). According to this notion, such Hpa2-binding protein(s) co-operate with HSPG (or function independent of HSPG) to mediate cell adhesion and signal transduction. If such a putative protein is highly abundant in cells (i.e., U87 glioma), and exhibits a very high affinity to Hpa2, than interaction with HS may become secondary and thus not affected by heparin. Alternatively, specific disaccharide composition, sequences and modifications of HS may underlie the differential responses to heparin. Studies towards these possibilities are underway. Interestingly, the phosphorylation of Erk, FAK, and Akt was increased prominently in cells plated on Hpa2, comparable in magnitude to their phosphorylation in cells plated on fibronectin (**Figure 3**). However, while typical adherent junctions were evident in cells plated on fibronectin, signifying classical integrin-mediated cell adhesion, cells plated on Hpa2 did not present such structures (**Figure 3C**), favoring HSPG-mediated adhesion (39, 54, 55).

Modulation of Erk phosphorylation by Hpa2 was observed in two other experimental settings. A marked increase of Erk phosphorylation was noted in Xenopus embryos in which Hpa2 expression was knocked-out vs control wt embryos, suggesting that in normal development Hpa2 attenuates Erk phosphorylation coinciding with early stages of embryo motility (56). Moreover, the protective effect of Hpa2 against LPS-mediated endothelial cell damage was associated with reduced Erk phosphorylation (47). This suggests that Hpa2 can modulate Erk phosphorylation up or down depending on the system and experimental setting being investigated. Notably, application of the 1C7 antibody and thus displacement of the protein from cell surface HS glycocalyx structures reversed the protective effect of Hpa2, resulting in strong activation of the LPS response (47). Collectively, these results suggest that Hpa2 modulates Erk phosphorylation, and this function likely involves cell surface HSPG. Thus, attenuated cell migration following exogenous addition of Hpa2 may result from decreased phosphorylation of Erk and related signaling molecules. More research is required to reveal the signaling cascades elicited by Hpa2, and studies focusing on this direction are currently underway.

Unexpectedly, exogenous addition of Hpa2 resulted also in the disruption of well-organized cell colonies. Moreover, exposure of the cell colonies to Hpa2 for only 15 (not shown) and 30 min was sufficient to elicit the scattering effect (**Figure 5B**, 30’), indicating that signaling, rather than gene transcription, is involved. At a first glance, the scattering effect looked surprising and counterintuitive to the notion of Hpa2 as a tumor suppressor (14–17). However, given the role of Hpa2 in development (56) and the tight coordination of cell motility during developmental processes, decreased (**Figure 4**) and enhanced (**Figure 5**) cell motility are two sides of the same coin. Thus, Hpa2 expression is likely tightly regulated. Mechanisms that regulate Hpa2 expression have not been elucidated yet and are currently under investigation.

Notably, the ability of Hpa2 to modulate cell adhesion, migration and scattering were reversed by anti-Hpa2 mAbs, thus laying confidence that these are truly effects of Hpa2. Importantly, mAb 20c5 was noted to reverse the effects of Hpa2 on cell adhesion (**Figure 2B**), cell migration (**Figure 4B**), and cell scattering (**Figure 6A**) (**Table 1**), suggesting that its epitope is critical for Hpa2 functions. Elucidating the sequence of this epitope employing peptide mapping or co-crystallization of Hpa2 with this and other anti-Hpa2 functional mAbs (i.e., 1c7) will enable the mapping of functional domains within the Hpa2 protein. This would greatly improve our understating of Hpa2 functional domains, including its HS-binding motif. Notably, HS-binding domains have been characterized in heparanase (57) but are not conserved in Hpa2 (58). Applying the ClusPro server, Coombe and Gandhi (58) modeled a predicted crystal structure of Hpa2 to the crystal structure of pro-heparanase (59). Interestingly the analysis revealed that the most favored region for binding heparin lies within the C-terminus of Hpa2 (58). More specifically, the residues predicted by the ClusPro docking analysis to mediate the binding of Hpa2 to HS were Gln^524^, Arg^466^, Arg^508^, Lys^509^, Lys^510^, Lys^512^, Arg^561^, Arg^564^, Arg^567^, and Thr^568^, different from previous prediction (13). Studies directed toward identifying functional domains of Hpa2 are currently in progress.

Taken together, we describe previously unrecognized functions of Hpa2, profoundly affecting cell adhesion and motility. Cell migration is critical in diverse biological processes, ranging from development to wound healing, metastasis, and immune responses, all of which require the orchestrated movement of cells in particular directions to specific locations. The capacity of Hpa2 to direct cell motility suggests that this protein plays a significant role in diverse biological settings, as critically emerged from the lethal phenotype of Hpa2-mutant mice (43, 60). These results strongly encourage an in-depth investigation of Hpa2, toward elucidating its role in health and disease.

## Data Availability Statement

The original contributions presented in the study are included in the article/**Supplementary Material**. Further inquiries can be directed to the corresponding author.

## Author Contributions

Conception and design: IV, NI. Development of methodology: MG-C, SF, GA. Acquisition of data: MG-C. Analysis and interpretation of data: MG-C, NI, IV. Writing, review, and/or revision of the manuscript: MG-C, NI, IV. Study supervision: IV. All authors contributed to the article and approved the submitted version.

## Funding

These studies were generously supported by research grants awarded by the Israel Science Foundation (grant 1021/19); the United States-Israel Binational Science Foundation (BSF); and the Israel Cancer Research Fund (ICRF). IV is a Research Professor of the ICRF.

## Conflict of Interest

The authors declare that the research was conducted in the absence of any commercial or financial relationships that could be construed as a potential conflict of interest.

## Supplementary Material

Supplementary Figure 1
**(A)** Purified Hpa2. **(B)** RPMI 8226 myeloma cells were plated on dishes coated with BSA, heparanase, or Hpa2. After 1 hour, the medium was removed and attached cells were visualized. Shown are representative images at x100 (original magnification). **(C, D)** Cell migration. Porcine aortic endothelial (PAEC, upper panels), SIHN-013 laryngeal carcinoma (second panels), and A549 lung carcinoma (third panels) cells were plated in ibidi cell migration inserts apparatus (Planegg, Germany) until confluent. The barrier was then removed, cell cultures were washed and changed to serum-free medium or medium containing 2% serum, and migration into the defined cell-free gap was inspected in the absence (Con, left) or presence of 10 µg/ml purified Hpa2 (right). Migration of non-transformed MCF10A human breast cells in the absence (Control) or presence of EGF (20 ng/ml) alone or EGF and Hpa2 is shown in **(C)**. Shown are representative photomicrographs taken 24 hours after the addition of Hpa2. **(E)** Cell scattering. MDCK cells were allowed to grow for 2-3 days until cell colonies were formed. Heparanase or Hpa2 (10 μg/ml) was then added and colonies morphology was examined after 24 hours vs control (Con) untreated cells. Shown are representative images at x100 (original magnification).
