# Supplementary figures and images for: Elucidating the Consequences of Heparan Sulfate Binding by Heparanase 2
Source: Front Oncol. 2021 Jan 29;10:627463. doi: 10.3389/fonc.2020.627463 (PMC7879983; doi:10.3389/fonc.2020.627463)

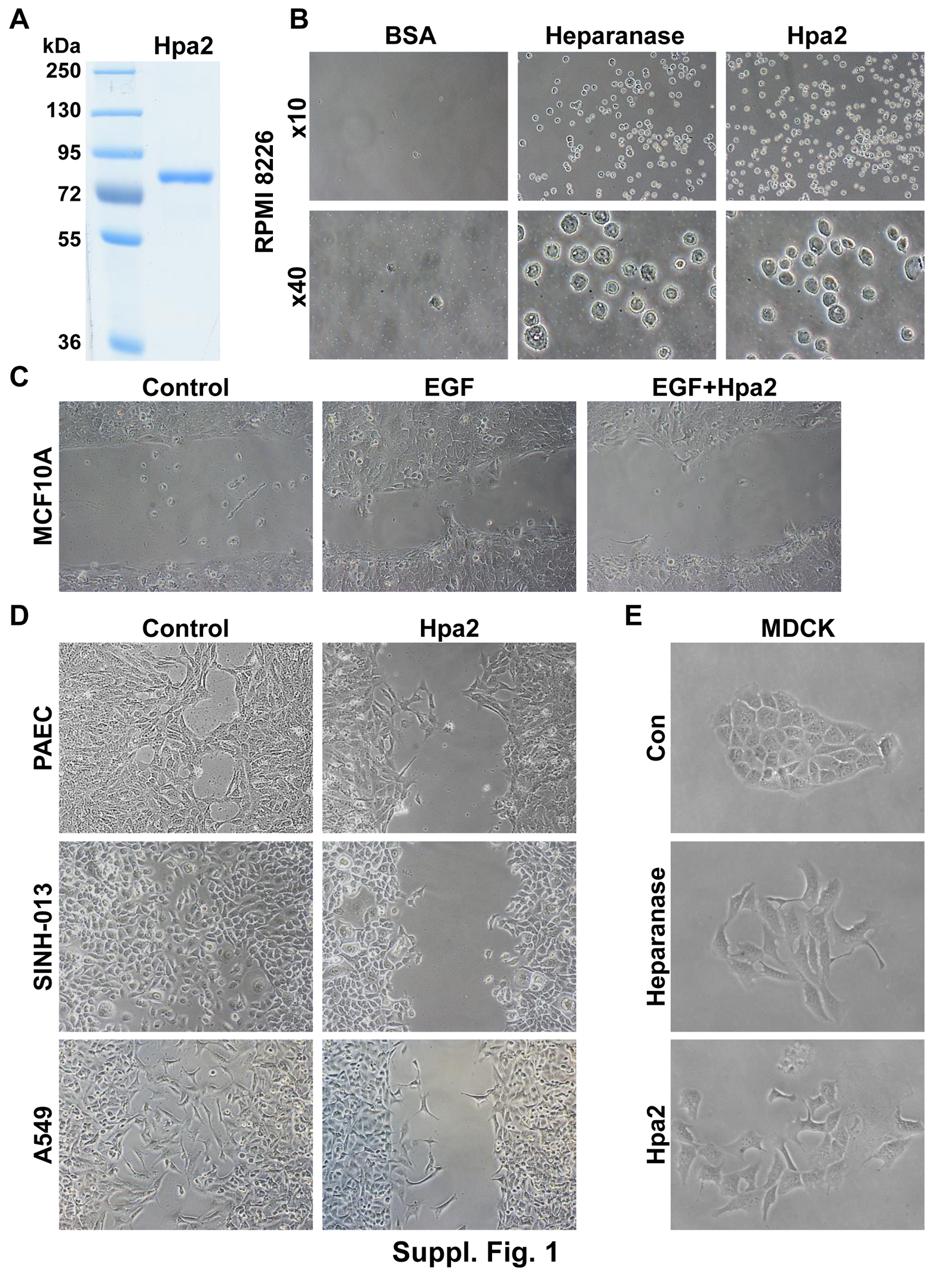

Supplement: Supplementary Figure 1 — (A) Purified Hpa2. (B) RPMI 8226 myeloma cells were plated on dishes coated with BSA, heparanase, or Hpa2. After 1 hour, the medium was removed and attached cells were visualized. Shown are representative images at x100 (original magnification). (C, D) Cell migration. Porcine aortic endothelial (PAEC, upper panels), SIHN-013 laryngeal carcinoma (second panels), and A549 lung carcinoma (third panels) cells were plated in ibidi cell migration inserts apparatus (Planegg, Germany) until confluent. The barrier was then removed, cell cultures were washed and changed to serum-free medium or medium containing 2% serum, and migration into the defined cell-free gap was inspected in the absence (Con, left) or presence of 10 µg/ml purified Hpa2 (right). Migration of non-transformed MCF10A human breast cells in the absence (Control) or presence of EGF (20 ng/ml) alone or EGF and Hpa2 is shown in (C). Shown are representative photomicrographs taken 24 hours after the addition of Hpa2. (E) Cell scattering. MDCK cells were allowed to grow for 2-3 days until cell colonies were formed. Heparanase or Hpa2 (10 μg/ml) was then added and colonies morphology was examined after 24 hours vs control (Con) untreated cells. Shown are representative images at x100 (original magnification). [file Image_1.tif]
